# The effect of the head-up position on cardiopulmonary resuscitation: a systematic review and meta-analysis

**DOI:** 10.1186/s13054-021-03797-x

**Published:** 2021-10-30

**Authors:** Cheng-Chieh Huang, Kuan-Chih Chen, Zih-Yang Lin, Yu-Hsuan Chou, Wen-Liang Chen, Tsung-Han Lee, Kun-Te Lin, Pei-You Hsieh, Cheng Hsu Chen, Chu-Chung Chou, Yan-Ren Lin

**Affiliations:** 1 Department of Emergency and Critical Care Medicine, Changhua Christian Hospital, 135 Nanshsiao Street, Changhua, 500 Taiwan; 2 Department of Biological Science and Technology, National Yang Ming Chiao Tung University, Hsinchu City, 30010 Taiwan; 3 School of Medicine, Kaohsiung Medical University, Kaohsiung, 807 Taiwan; 4 School of Medicine, Chung Shan Medical University, Taichung, 402 Taiwan; 5 College of Medicine, National Chung Hsing University, Taichung, 402 Taiwan

**Keywords:** Head-up position, Cardiopulmonary resuscitation, Cerebral perfusion pressure

## Abstract

**Objective:**

Experimental studies of head-up positioning (HUP) during cardiopulmonary resuscitation (CPR) have had some degree of conflicting published results. The current study aim was to analyze and reconcile those discrepancies in order to better clarify the effects of HUP CPR compared to conventional supine (SUP) CPR.

**Methods:**

Three databases (PubMed, EMBASE and Cochrane Library) were searched comprehensively (from each respective database's inception to May 2021) for articles addressing HUP CPR. The primary outcome to be observed was cerebral perfusion pressure (CerPP), and secondary outcomes were mean intracranial pressure (ICP), mean arterial pressure (MAP), coronary perfusion pressure (CoPP) and frequencies of return of spontaneous circulation (ROSC).

**Results:**

Seven key studies involving 131 animals were included for analysis. Compared to SUP CPR, CerPP (MD 10.37; 95% CI 7.11–13.64; *p* < 0.01; *I*^2^ = 58%) and CoPP (MD 7.56; 95% CI 1.84–13.27, *p* = 0.01; *I*^2^ = 75%) increased significantly with HUP CPR, while ICP (MD − 13.66; 95% CI − 18.6 to –8.71; *p* < 0.01; *I*^2^ = 96%) decreased significantly. Combining all study methodologies, there were no significant differences detected in MAP (MD − 1.63; 95% CI − 10.77–7.52; *p* = 0.73; *I*^2^ = 93%) or frequency of ROSC (RR 0.9; 95% CI 0.31–2.60; *p* = 0.84; *I*^2^ = 65%). However, in contrast to worse outcomes in studies using immediate elevation of the head in a reverse Trendelenburg position, study outcomes were significantly improved when HUP (head and chest only) was introduced in a steady, graduated manner following a brief period of basic CPR augmented by active compression–decompression (ACD) and impedance threshold (ITD) devices.

**Conclusion:**

In experimental models, gradually elevating the head and chest following a brief interval of circulatory priming with ACD and ITD devices can enhance CoPP, lower ICP and improve CerPP significantly while maintaining MAP. This effect is immediate, remains sustained and is associated with improved outcomes.

**Supplementary Information:**

The online version contains supplementary material available at 10.1186/s13054-021-03797-x.

## Introduction

Cardiac arrest is the most critical challenge faced by every clinical physician because it has varying etiologies and a high mortality rate [1]. High-quality cardiopulmonary resuscitation (CPR) and improvement in the emergency medical service (EMS) system have been proven to result in a higher return of spontaneous circulation (ROSC) rate in out-of-hospital cardiac arrest (OHCA) patients, and the survival rate has improved over time since 2006. However, less than 10% of patients survive to hospital discharge, and survival with good neurological outcomes is even lower [2–4].

Insufficient brain perfusion is a key factor in poor neurological outcomes. Cerebral perfusion pressure (CerPP), which is calculated as the mean arterial pressure (MAP) minus the intracranial pressure (ICP), decreases dramatically during cardiac arrest for the following reasons. First, once cardiac arrest occurs, inflammatory systems are activated to respond to whole-body ischemia, which results in increased membrane permeability [5]. In addition, the blood–brain barrier (BBB) breaks down because of intracellular acidosis, stopping oxidative phosphorylation and leading to accumulation of lactate [6]. Due to these two effects, serum proteins and water pass from the blood to brain tissue, which leads to neuronal, glial or axonal injuries [7] and increases ICP. Second, chest compressions are performed to try to expel blood out of the heart and create forward flow, but also cause increased thoracic pressure and impede venous return [8]. Finally, optimal CPR can provide approximately 20–30% of pre-arrest cardiac output [9], and only 30% of it flows to the brain [6]. As a result, the rate of survival with good neurological outcomes is dismal.

Compared to conventional CPR (CCPR), active compression–decompression (ACD) CPR with an impedance threshold device (ITD) has been shown to improve the ROSC rate and survival to discharge with favorable neurological outcomes [10–12]. Theoretically, ACD, by an upwards lifting force during the decompression phase, could decrease intrathoracic pressure, leading to augmented venous return. ITD could cause the same effect by selectively restricting airflow into the lungs during the decompression phase. However, a recent meta-analysis that focused on ACD with/without ITD CPR and CCPR revealed similar ROSC rates [13].

In recent years, animal studies have revealed the head-up positions (HUP) with ACD + ITD CPR or automated (LUCAS 2.0) + ITD CPR could decrease ICP and improve CerPP [14] and that it may even improve coronary perfusion pressure (CoPP) [15] in animal experiments. As to HUP, some studies tilted whole body up, also called “the reverse-Trendelenburg position,” to lower ICP. However, this position could also decrease MAP due to pooling more blood in lower extremities [15, 16]. To avoid this condition, other studies elevated the “head and chest up only” [14, 17, 18]. No matter what HUP posed, compared to CCPR, in which the patient lies down at 0 degrees, elevating the head during CPR could accelerate brain venous return and the hydrostatic displacement of cerebrospinal fluid (CSF) from the cerebral ventricles to the spinal cavity [19]. Thus, ICP decreases and CerPP increases. In addition, facilitating venous return may increase CoPP, and a higher CoPP is associated with a higher ROSC rate [20]. Furthermore, with the combination of HUP ACD + ITD CPR, a recent animal study found that controlled sequential elevation (CSE) rather than a specific head-up angle could maximize CerPP and improve the ROSC rate and neurological outcome [21]. However, Park et al. [22] demonstrated that head-up CPR could worsen the survival rate. HUP might be considered a “next-step” intervention in the ICP-lowering/CPP-enhancing paradigm. Therefore, some of the preclinical data regarding HUP CPR appear to be mixed, thus prompting us to better explore and clarify the effect of HUP CPR. In this article, a comprehensive systematic review and meta-analysis was performed to evaluate the effect of HUP CPR in animal models and to draw conclusions to establish a new strategy for CPR in the future.

## Method

We conducted this study according to the Cochrane Handbook for Systematic Reviews of Interventions guidelines [23], Hooijmans et al. [24] and the preferred reporting items for systematic reviews and meta-analyses (PRISMA) statements [25].

### Eligibility criteria

#### Types of studies

All types of studies were eligible except for case reports, reviews, abstract publications and conference presentations because there is no detailed study design to assess quality or data to analyze.

#### Types of subjects

We included all types of animal studies. If different animal species were included, then we analyzed and discussed them separately.

#### Types of interventions

Studies comparing HUP CPR with supine position (SUP) CPR were included. There were no restrictions on the head-up degree, no-flow time, CPR device, CPR protocol or CPR duration. The head-up degree is defined as the angle between the head–chest plane and the horizontal plane. A head-up degree of zero means the supine position. The no-flow time was defined as the time from cardiac arrest to the start of CPR. CPR duration was defined as the time from the start of CPR to the stopping point of CPR, regardless of whether ROSC was achieved.

#### Types of outcome measures

The primary outcome was the CerPP in both groups. The secondary outcomes were the mean ICP, MAP, CoPP and ROSC rates. If the studies presented outcomes such as systolic pressure, diastolic pressure and ICP during the compression/decompression phase, then we estimated MAP as (systolic pressure + diastolic pressure)/2 and mean ICP as (ICP during compression + ICP during decompression)/2 because the compression time is approximately equal to the decompression time during CPR.

### Search methods for the identification of studies

Studies were obtained by comprehensively searching 3 databases (PubMed, EMBASE and the Cochrane Library) from database inception to May 10, 2021 without language restriction. The following key words or medical subject heading (MeSH) terms were used: head-up position or head up or torso up or head elevation or tilt AND resuscitation or CPR. We also reviewed the references of the identified articles to avoid missing possible articles.

### Data extraction and quality assessment

Two authors (C.K. Chen and Z.Y. Lin) searched articles from 3 databases and extracted the data independently. We collected the following information from each eligible study: authors; publication year; study design; study group; CPR protocol; CPR device; no-flow time; CPR duration; intervention/control details; and outcome data. If the data were presented in a graph instead of as digits, then we used GetData Graph Digitizer software, version 2.26 (http://getdata-graph-digitizer.com/download.php), to extract the data.

The Animal Research: Reporting of In Vivo Experiments (ARRIVE) guidelines 2.0 [26] were used to evaluate the quality of the included articles, as assessed by two authors (C.K. Chen and Z.Y. Lin) independently. There were 21 domains that were assessed for each study. Each domain was rated as “+” if it met the domain recommendation; otherwise, it was rated as “−.”

Finally, any disagreement that occurred during the article search, data extraction or quality assessment was resolved by a discussion between a third author (C.C. Huang) and the two previously mentioned authors to reach a consensus.

### Statistical analysis

We used Review Manager (RevMan [Computer program], version 5.4.1, The Cochrane Collaboration, 2020) to analyze all data. Because some of the included studies presented results as the standard error of the mean (SEM), we converted SEM to the standard deviation (SD) by the formula SEM = SD/√(sample size) [27]. We expressed continuous data as mean differences (MDs) and dichotomous data as risk ratios (RRs). In addition, 95% confidence intervals (CIs) were calculated for all of the results.

We applied a random-effects model for all analyses and Chi-square and *I*^2^ tests to evaluate heterogeneity. A subgroup analysis was conducted based on the change in position in CPR and the CPR duration of fixed-position CPR. To evaluate other possible factors that could affect CPR qualities, we further performed a subgroup analysis as follows: 1. Automated + ITD CPR versus ACD + ITD CPR; 2. “priming” versus “no priming”; and 3. “head/chest up only” position versus "the reverse-Trendelenburg position". In addition, we performed a visual inspection of the funnel plot to assess publication bias, and a sensitivity analysis was conducted by repeating the analysis after removing one study at a time. Finally, the results were presented in forest plots.

## Results

**Fig. 1:**
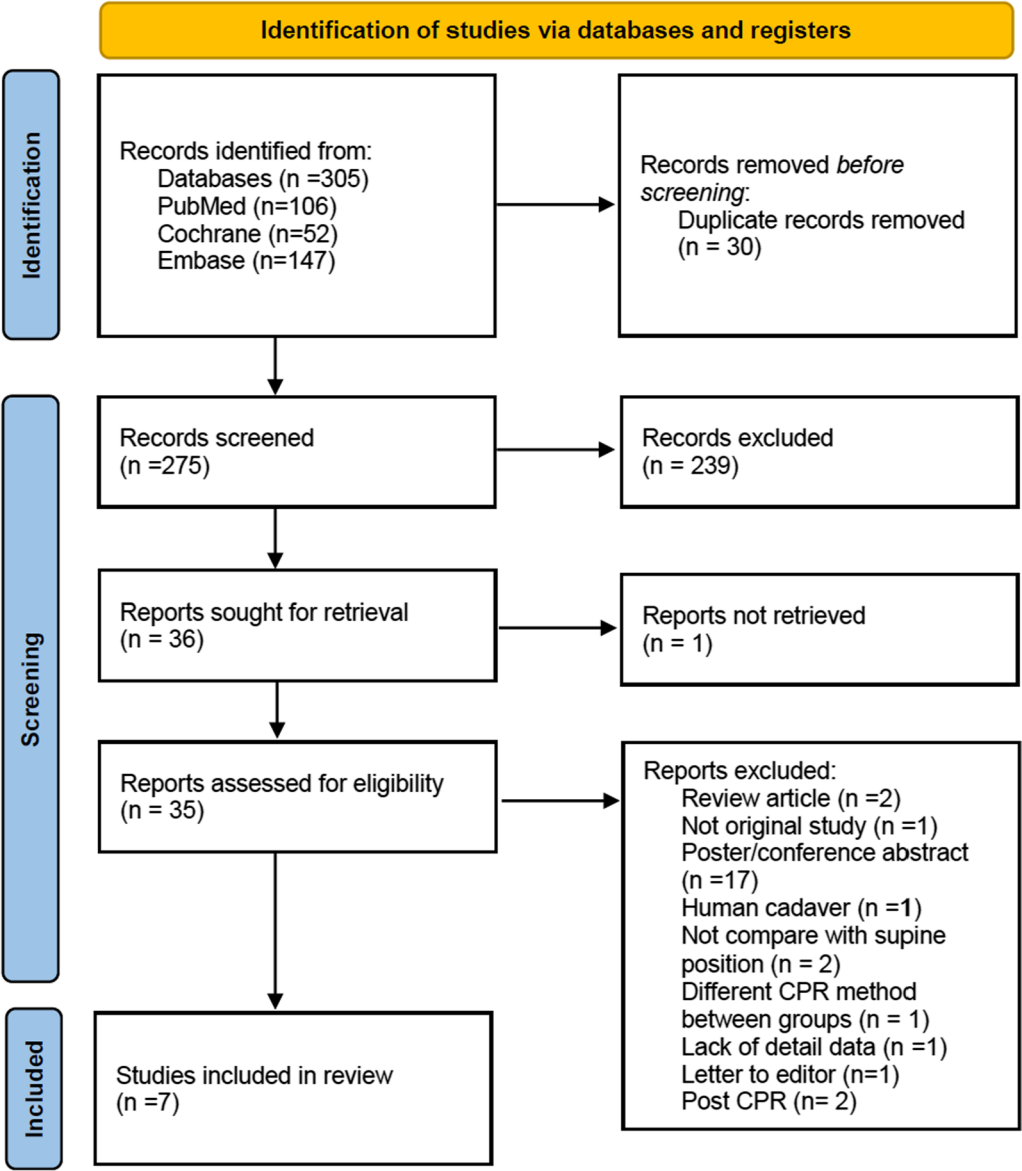
PRISMA flow diagram

A total of 305 studies were initially retrieved from 3 databases (PubMed: 106, EMBASE: 147, Cochrane: 52). After removing duplicates (*n* = 30), excluding 239 articles by screening the titles and abstracts and 1 records not retrieved, 35 full-text articles were assessed for eligibility. Next, we excluded 27 articles (because they were review articles, not original studies, or posts/conference abstracts, studied human cadavers, not compare with supine position, lacked detailed data, letter to editor or post CPR). Initially, eight studies [14–18, 21, 22, 28], including 147 pigs, were included; however, we excluded one study (that used different CPR methods with different angles) because of the lack of a comparison group [21]. Therefore, 7 studies with 131 subjects were ultimately analyzed in the meta-analysis (Fig. 1).

The head-up angle ranged from − 60° to 60°. Three studies [15, 16, 22] used the Trendelenburg position or reverse-Trendelenburg position, and the others elevated the head and shoulders at only 30°. Thus, we presented our outcomes by comparing the head-up position at 30° to the supine position. The no-flow time ranged from 6 to 15 min, and the CPR time ranged from 6 to 26 min. All of the included studies used automated (Lucas chest compress system) or ACD CPR with/without ITD, and two of them [14, 18] also used CCPR (Table 1).

**Table 1:** The characteristics of the included studies

Study, year	Design	Species	Sample size	No-flow time	CPR method	Head-up angle	CPR time
Debaty et al. [15]	Non-RCT	Female Yorkshire farm pigs	22	6 min	LUCAS 2.0 + ITD CPR	− 30°–50°^a^	22 min
Ryu et al. [18]	RCT	Female Yorkshire farm pigs	30	8 min	CCPR, ACD + ITD CPR^c^	0° and 30°	22 min
Kim et al. [16]	Non-RCT	Female pigs	12	6 min	LUCAS 2.0 + ITD CPR	− 60°–60°^a^	18 min
Moore et al. [17]	RCT	Female Yorkshire farm pigs	18	8 min	ACD + ITD CPR^c^	0° and 30°	20 min
Putzer et al. [28]	RCT	Domestic pigs	20	8 min	LUCAS 2.0 CPR	0° and 30°	20 min
Moore et al. [14]	Non-RCT	Female Yorkshire farm pigs, pig cadaver, human cadaver	27^b^	6 min	CCPR, ACD + ITD CPR^cd^	0° and 30°	26 min
Park et al. [22]	RCT	Female Yorkshire farm pigs	18	15 min	LUCAS 2.0 + ITD CPR	0° and 30°^a^	6 min

*RCT* randomized controlled trial, *ACD* active compression–decompression, *CPR* cardiopulmonary resuscitation, *ITD* impedance threshold device, *CCPR* conventional cardiopulmonary resuscitation

^a^A negative number represents the Trendelenburg position, and a positive number represents the reverse-Trendelenburg position

^b^Includes 9 human cadavers

^c^Use of a customized animal ACR-CPR machine

^d^Use of a manual ACD pump + ITD (ResQPump) on human cadavers

### Quality assessment

The quality assessments of our included studies are shown in Table 2. The average score was 16.86 ± 0.83 (mean ± SD) and ranged from 15 to 18. A visual inspection of the funnel plot revealed symmetry, which suggested no publication bias (Additional file 1).

**Table 2:** Quality assessments according to the ARRIVE guidelines 2.0

	1	2	3	4	5	6	7	8	9	10	11	12	13	14	15	16	17	18	19	20	21	Quality score
Debaty 2015	+	+	−	+	+	+	+	+	+	+	+	+	+	+	−	−	+	−	+	+	+	17
Ryu 2016	+	+	−	+	−	+	+	+	+	+	+	+	+	+	−	+	+	+	+	−	+	17
Kim 2017	+	+	−	+	+	+	+	−	+	+	+	+	+	+	−	−	+	+	+	+	+	17
Moore 2017	+	+	−	+	+	+	+	+	+	+	+	+	+	+	−	−	+	+	+	+	+	18
Putzer 2018	+	+	−	+	−	+	+	−	+	+	+	+	+	+	−	+	+	+	+	+	+	17
Moore 2018	+	−	−	−	−	+	+	+	+	+	+	+	+	+	−	+	+	+	+	−	+	15
Park 2019	+	+	−	+	−	+	+	+	+	+	+	+	+	+	−	+	+	+	+	−	+	17

Quality items: (1) study design; (2) sample size; (3) inclusion and exclusion criteria; (4) randomization; (5) blinding; (6) outcome measures; (7) statistical methods; (8) experimental animals; (9) experimental procedures; (10) results; (11) abstract; (12) background; (13) objectives; (14) ethical statement; (15) housing and husbandry; (16) animal care and monitoring; (17) interpretation/scientific implications; (18) generalizability/translation; (19) protocol registration; (20) data access; and (21) declaration of interests

### Primary outcome

**Fig. 2:**
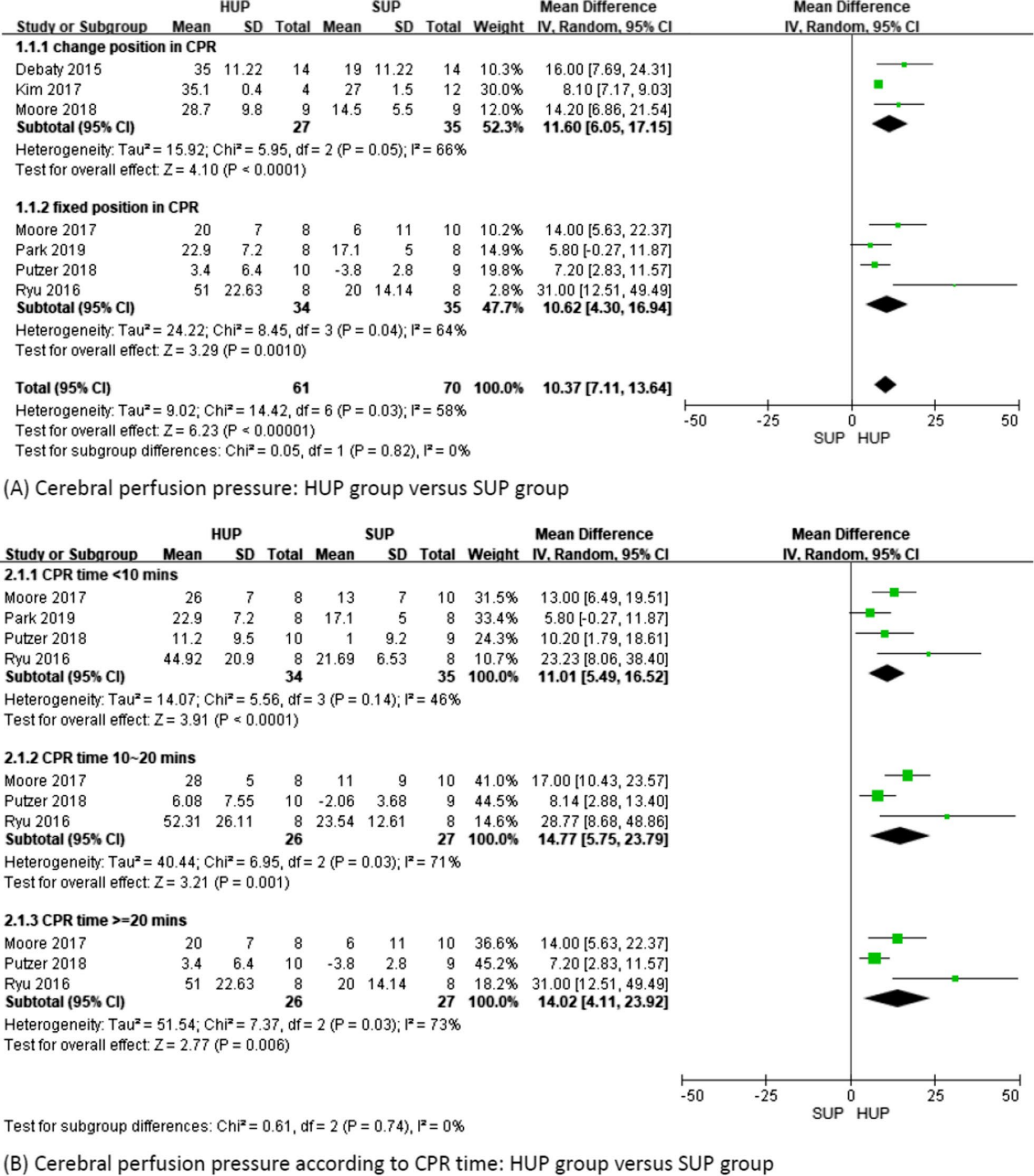
Cerebral perfusion pressure

Seven studies including 131 subjects were analyzed. The overall CerPP increased significantly in the HUP group compared to the SUP group (MD 10.37; 95% CI 7.11–13.64; *p* < 0.01; I^2^ = 58%) (Fig. 2A). The subgroup analysis of CerPP showed that regardless of whether the position was changed during CPR, CerPP was significantly increased in the HUP group (MD 11.6; 95% CI 6.05–17.15; *p* < 0.01; I^2^ = 66% and MD 10.62; 95% CI 4.3–16.94; *p* < 0.01; I^2^ = 64%) (Fig. 2A).

In addition, in fixed-position CPR studies, we further evaluated whether the duration of CPR affected our primary outcome, and the results still showed a significant increase in CerPP in the HUP group, regardless of whether the duration of CPR was < 10 min, 10–20 min, or > 20 min (*p* < 0.01 in all 3 subgroups) (Fig. 2B).

### Secondary outcomes

#### ICP and MAP

**Fig. 3:**
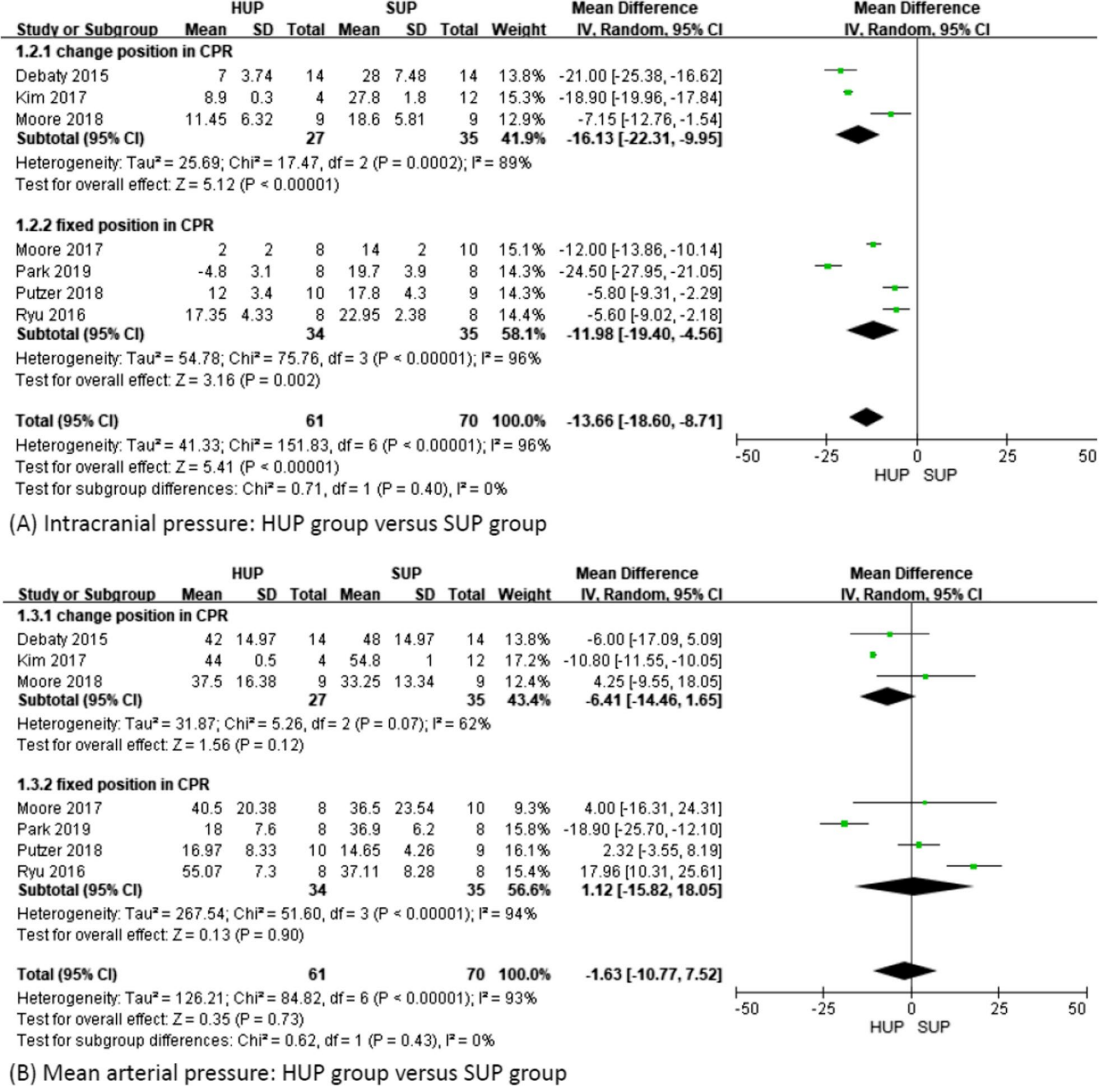
Intracranial pressure and mean arterial pressure

The overall ICP was significantly lower in the HUP group than in the SUP group (MD − 13.66; 95% CI − 18.6 to − 8.71; *p* < 0.01; *I*^2^ = 96%), regardless of whether the position was changed during CPR (MD − 16.13; 95% CI − 22.31 to − 9.95; *p* < 0.01; *I*^2^ = 89% and MD − 11.98; 95% CI − 19.4 to − 4.56; *p* < 0.01; *I*^2^ = 96%) (Fig. 3A) or the duration of CPR (*p* = 0.03 in CPR time < 10 min and *p* < 0.01 in both remaining subgroups) (Additional file 2). However, we did not find a significant difference in MAP between the HUP group and the SUP group (MD − 1.63; 95% CI − 10.77–7.52; *p* = 0.73; *I*^2^ = 93%), regardless of whether the position was changed during CPR (MD − 6.41; 95% CI − 14.46–1.65; *p* = 0.12; *I*^2^ = 62% and MD 1.12; 95% CI − 15.82–18.05; *p* = 0.9; *I*^2^ = 94%) (Fig. 3B) or the duration of CPR (Additional file 3).

### CoPP

**Fig. 4:**
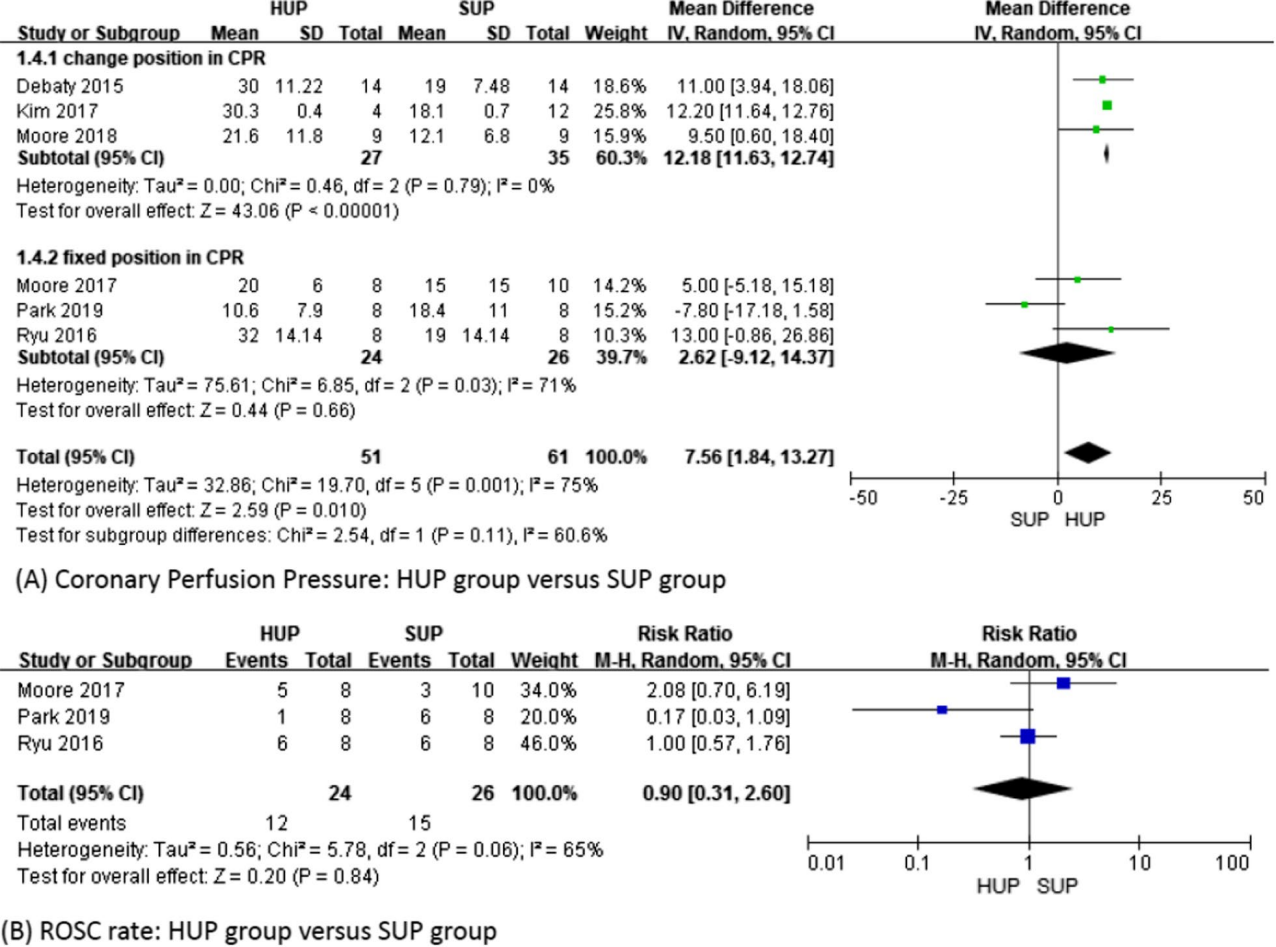
Coronary perfusion pressure and ROSC rate

CoPP also increased significantly in the HUP group (MD 7.56; 95% CI 1.84–13.27; *p* = 0.01; *I*^2^ = 75%). However, the subgroup analysis revealed that CoPP was increased in the HUP group when the position during CPR was changed (MD 12.18; 95% CI 11.63–12.74; *p* < 0.01; *I*^2^ = 0%), while a fixed position during CPR was not associated with an increase in CoPP (MD 2.62; 95% CI − 9.12–14.37; *p* = 0.66; *I*^2^ = 71%) (Fig. 4A). We did not observe a difference between the groups according to the duration of CPR (Additional file 4).

### ROSC rate

Only 3 studies [17, 18, 22] with 50 subjects reported the ROSC rate. The ROSC rate was 50% (12/24) in the HUP group and 58% (15/26) in the SUP group. There was no significant difference between the groups (RR 0.9; 95% CI 0.31–2.60; *p* = 0.84; *I*^2^ = 65%) (Fig. 4B).

### Subgroup analysis

In comparing automated + ITD CPR vs. ACD + ITD CPR, the ACD + ITD CPR group had higher CerPP, MAP and ICP (all *p* < 0.05). CoPP revealed similarities between the CPR methods (*p* = 0.67) (Additional file 5). In comparing “priming” versus “no priming,” the “priming” group had higher CerPP and CoPP (all *p* < 0.05). ICP and MAP were similar in both groups (*p* = 0.83 and *p* = 0.44, respectively). However, once we removed Putzer et al. [28] because of the markedly low MAP in SUP (less than 40% compared to the other studies, and the result was consistent with the CCPR result in a previous study [18]), MAP was significantly higher in the “priming” group (*p* < 0.01) (Additional file 6). In comparing “head/chest up only” position vs. the reverse-Trendelenburg position, the “head/chest up only” position group had higher ICP and MAP (all *p* < 0.01). CerPP and CoPP were similar in both groups (*p* = 0.26 and *p* = 0.67, respectively) (Additional file 7).

## Discussion

The most important result of our study is that HUP at 30° during CPR can significantly increase CerPP, mainly by reducing ICP, compared to SUP. CerPP, which is calculated by MAP minus ICP, could be increased by higher MAP and/or lower ICP. The reason for the lower ICP is that when elevating the head and body up to 30° during CPR, ICP will decrease by facilitating brain venous return and CSF movement into the spinal subarachnoid space, which is consistent with previous studies, even at different elevation angles ranging from 10° to 50° [15]. In addition, the reduction in ICP also decreases the resistance to forward brain blood flow, which is generated by each chest compression. This effect could explain the findings of 2 of our included studies [15, 17] that demonstrated that brain blood flow increased significantly in HUP compared with SUP. Furthermore, due to the heterogeneous study protocols in our included studies, we analyzed CerPP regarding whether the position during CPR was changed or fixed, and in the fixed position CPR studies, we further evaluated the duration of CPR, which showed significantly increased CerPP in all HUP groups. Thus, HUP CPR can reduce ICP when the head is elevated during CPR, and the effect could last the entire CPR duration.

The other important result is that we did not find a significant difference in MAP between the HUP and SUP groups, regardless of whether the position was changed during CPR or the duration of CPR. Maintaining sufficient blood pressure by pumping upwards to the brain is important in CPR. From a physiological perspective, elevating the head and chest in CPR may reduce MAP because of the gravity effect. Each chest compression will pump more “uphill” than in the supine position. However, ACD-CPR with ITD could generate sustained aortic pressure, and HUP may reduce the resistance of blood flow to the brain. Therefore, the net effect of MAP revealed no significant difference between the 2 groups in our study. The absolute MAP value was much lower in Putzer et al. [28], who did not use ITD in automated CPR, and MAP along with CerPP decreased gradually over time. Debaty et al. [15] revealed that MAP and CerPP showed a significant decrease immediately once ITD was removed in HUP CPR. These results could support our inferences. In addition, of our included studies, two [16, 16, 22] showed decreased MAP in HUP, while the others revealed no significant difference between the 2 groups. Both of them were in the reverse-Trendelenburg position rather than in the “head and chest up only” position. Because more blood deposits in the lower extremities, we speculate that just ACD-CPR with ITD may not overcome the physiological effect of the reverse-Trendelenburg position, which results in a decrease in MAP. Interestingly, pulmonary edema is a common complication of cardiac arrest [29]. Elevating the chest may have better blood-gas exchange caused by reduced lung congestion and pulmonary vascular resistance because of the gravity effect [18]. This potential benefit should be confirmed by more studies.

We also found higher CoPP in the HUP group than in the SUP group. CoPP is calculated by diastolic aortic pressure minus right atrial pressure [30]. Theoretically, while the head and chest are elevated, right atrial pressure is also decreased by the gravity effect. As a result, CoPP could be increased under ACD-CPR with ITD to maintain sufficient diastolic aortic pressure. Kim et al. [16] revealed that CoPP increased gradually from the head-down position and supine position to the head-up position and reached the highest CoPP at 30°. In contrast, the CoPP and MAP in Park et al. [22] were much lower than those in the other included studies. The methodology in this study, which is different from the others, did not overcome the effects of gravity for the following reasons: 1. “Not priming the cardio-cerebral circuit” at SUP before head elevation; 2. elevating the head too fast before CPR; 3. maintaining the reverse-Trendelenburg position at a steep angle for a prolonged period; and 4. using a mechanical device that may not provide an optimal ACD effect. However, two of the included studies [15, 17] directly measured heart flow and revealed no significant difference between the 2 groups. The calculated pressure does not translate directly to actual flow. Thus, further studies need to be conducted to clarify this point.

Only 3 included studies [17, 18, 22], including 50 Yorkshire farm pigs, reported the ROSC rate, and the results showed no difference between the two groups. In these 3 studies, Park et al. [22] revealed that the ROSC rate and survival rate were reduced significantly in the HUP group (ROSC rate: 1/8 in HUP vs. 6/8 in SUP, *p* = 0.04; survival rate: 0/8 in HUP vs. 6/8 in SUP), while the others [17, 18] showed no difference. Effective chest compressions with sufficient CoPP are crucial for successful CPR. In addition to the previously mentioned reasons that cause decreased MAP and CoPP, the 15-min untreated ventricular fibrillation (VF) time was also far longer than the others. These reasons could explain the dismal ROSC rate in HUP. Furthermore, due to the heterogeneous study design, we removed this study for analysis, and the result remained unchanged (RR 1.27; 95% CI 0.61–2.65; *p* = 0.53; *I*^2^ = 39%).

The overall impact of HUP is that it is now a complementary and pivotal component of the combined ICP lowering approach. HUP alone is not associated with a better outcome. Thus, there are some important findings in addition to HUP CPR. First, by using ACD + ITD in HUP CPR, both ACD and ITD could decrease intrathoracic pressure in the decompression phase, which leads to augmented venous return. HUP could also accelerate brain venous return by the gravity effect. More importantly, combining HUP CPR with both ACD and ITD might not appear to have additive effects but rather to produce a markedly synergistic effect in terms of near-normalizing (sustaining CerPP). These three important components are “interdependent” and have a high synergistic effect that could significantly improve CerPP [17, 18]. These "interdependent" components should not be lost in all of this. In contrast, two of the included [15, 28] studies revealed an absolutely lower MAP while not using ITD in HUP CPR. Another study revealed that the CerPP in SUP ACD + ITD CPR is higher than that in HUP CPR, which suggests that ACD + ITD is more effective in increasing CerPP than HUP alone [18]. In addition, our subgroup analysis revealed that ACD + ITD CPR had better CerPP than automated + ITD CPR. Second, the “priming step” (CPR in the supine position for minutes before HUP) is very important, and raising the head immediately without a priming step would relate to poor outcomes [31]. From a pathophysiological view, humans lose vessel autoregulation during cardiac arrest. Once we elevate the head/chest up before CPR in the supine position, blood will deposit in the lower body. Chest compressions to pump blood “uphill” will not overcome this effect in the short term, which results in disappointing outcomes. All of our included studies elevated the head/chest in the short term, but with the “priming step,” the CerPP was still higher in the HUP group than in the SUP group. In contrast, two of our inclusion studies [22, 28] rose subjects immediately after VF had a markedly lower MAP, CerPP (in Putzer et al. [28]) and CoPP (in Park et al. [22]). After removing these two studies (one study at a time), the overall results remained unchanged, which suggests that there were other factors that affected MAP. The current study [32] revealed that the “priming step,” 2 min of relative SUP CPR, had significantly higher CerPP than elevating head/chest immediately after 8 min of untreated VF in an animal model. Our subgroup analysis also supports this inference. With the “priming” step, CerPP, MAP and CoPP increased significantly when compared to “no priming.” In addition, “no priming” deceased the “absolute” MAP value, which means that it caused a lower MAP than SUP. Moreover, performing a dispatch-assisted or bystander SUP CPR until an EMS crew takes over is closer to reality (patients might not receive HUP CPR immediately after going into arrest). Third, the “head/chest up only” position could be superior to the reverse-Trendelenburg position. Due to the gravity effect, the reverse-Trendelenburg position will deposit more blood in the lower extremities. Although CerPP is similar in both positions, the reverse-Trendelenburg position also decreased the “absolute” MAP value found by our subgroup study. Fourth, within 2–4 min of CSE, instead of the absolute HUP angle or rapid elevation, CerPP achieves 50% of baseline in 2.5 min and over 80% in 7 min under CSE HUP ACD + ITD CPR, as noted by a recent study [31, 32]. Although the mechanism of CSE needs further research, Moore et al. [21] revealed that HUP CPR had good neurologic survival outcomes in an animal model after combining 4 factors (using ACD + ITD CPR, the “priming” step, elevated head/chest up only and CSE). Overall, we considered that the “priming timing,” “sequencing of HUP” and “using ACD and ITD” during resuscitation are better able to achieve favorable neurological outcomes than just elevating the head and chest. Every factor is very important, and it would be dangerous if any one factor is ignored.

In recent years, Pepe et al. [33] revealed a great ROSC rate improvement from 17.8 to 34.2% (*p* < 0.0001) after applying the CPR bundle on the EMS system in OHCA patients. This bundle combined HUP automated + ITD CPR, “priming” the cardio-cerebral circuit at SUP, the reverse Trendelenburg position and gradually elevating the patient up to 20° (somewhat like CSE), which resulted in a hopeful outcome. Furthermore, a recent study also points out that the priming step and CSE are both important for better outcomes [31].

The performance of HUP CPR continues to evolve and has been further refined compared to what was initially reported and analyzed by the previous studies mentioned above. In addition, many proven key factors are more important to improve ROCS rates and neurological outcomes, such as early activated EMS, defibrillation and high-quality CPR. Thus, HUP ACD + ITD CPR may be considered in specific situations (i.e., non-VF cases with OHCA) once this concept is applied to human CPR in the future.

To the best of our knowledge, this is the first meta-analysis that compares HUP CPR to SUP CPR in animal models. The strength of this analysis includes further confirming the effect of HUP CPR. In addition, we performed multiple subgroup analysis due to heterogeneous study protocols. Moreover, two authors used the ARRIVE guidelines 2.0 to evaluate the quality of the included studies.

### Limitations

There were also some limitations. First and most importantly, the results of our study may not be totally transferrable to humans. All of the included studies used “healthy” animals, and a ventricular fibrillation model was used to simulate cardiac arrest. However, unlike animal experiments, cardiac arrest is caused by more complex factors and has more etiologies in humans. Most initial rhythms of cardiac arrest are non-shockable, both in in-hospital cardiac arrest and in out-of-hospital cardiac arrest, which accounts for 80% [34, 35], and human CPR physiology is more dynamic. In addition, until now, only one study [14] revealed that human cadavers had similar changes in the ratio of blood flow in HUP with ACD-CPR despite many different anatomical structures between humans and swine models. Further investigations that focus on HUP CPR physiology in human cadavers are warranted. Therefore, the outcome in this manuscript should not translate completely to humans before larger sample size and well-designed studies are published. Second, most of the included studies (5 over 7) were performed in the same laboratory with the same team. This could lead to single-source bias. Third, all of the included studies used calculated CerPP and CoPP. Only 2 studies further measured brain blood flow and heart blood flow directly by using microspheres. Although high perfusion pressure is associated with high blood flow, the evidence is indirect rather than direct. Finally, even though our study demonstrates strong evidence of increasing CerPP by lower ICP in HUP CPR, it is still unclear whether this benefit could equal an increased survival rate with good neurological outcome. Thus, further large-sample and standardized research is essential to confirm the optimal resuscitation protocols for humans and animals.

## Conclusion

In experimental models, gradually elevating the head and chest following a brief interval of circulatory priming with ACD and ITD devices can enhance CoPP, lower ICP and improve CerPP significantly while maintaining MAP. This effect is immediate, remains sustained and is associated with improved outcomes. This study further confirms the benefit of HUP ACD + ITD CPR in animal models, and further large-sample and standardized research is warranted to clarify the optimal resuscitation protocols for humans as well as animals.

## Supplementary Information

**Additional file 1.** The funnel plot.

**Additional file 2.** ICP in different CPR time.

**Additional file 3.** MAP in different CPR time.

**Additional file 4.** CoPP in different CPR time.

**Additional file 5.** Automated + ITD versus ACD + ITD CPR.

**Additional file 6.** “Priming” versus “no priming”.

**Additional file 7.** “Head/chest up only” position versus “reverse-Trendelenburg” position

## Data Availability

The datasets used and/or analyzed during the current study are available from the corresponding author on reasonable request.

## Abbreviations

HUP: Head-up position
SUP: Supine position
CPR: Cardiopulmonary resuscitation
CerPP: Cerebral perfusion pressure
ICP: Intracranial pressure
MAP: Mean arterial pressure
CoPP: Coronary perfusion pressure
ROSC: Return of spontaneous circulation
ACD: Active compression–decompression
ITD: Impedance threshold device
EMS: Emergency medical service
OHCA: Out-of-hospital cardiac arrest
BBB: Blood–brain barrier
CSF: Cerebrospinal fluid
PRISMA: Preferred reporting items for systematic reviews and meta-analyses
MeSH: Medical subject heading
ARRIVE: Animal research: reporting of in vivo experiments
SEM: Standard error of the mean
SD: Standard deviation
RR: Risk ratios
CI: Confidence intervals
VF: Ventricular fibrillation
CSE: Controlled sequential elevation
CCPR: Conventional cardiopulmonary resuscitation
