# Supplementary figures and images for: The effect of the head-up position on cardiopulmonary resuscitation: a systematic review and meta-analysis
Source: Crit Care. 2021 Oct 30;25:376. doi: 10.1186/s13054-021-03797-x (PMC8557496; doi:10.1186/s13054-021-03797-x)

Additional file 1


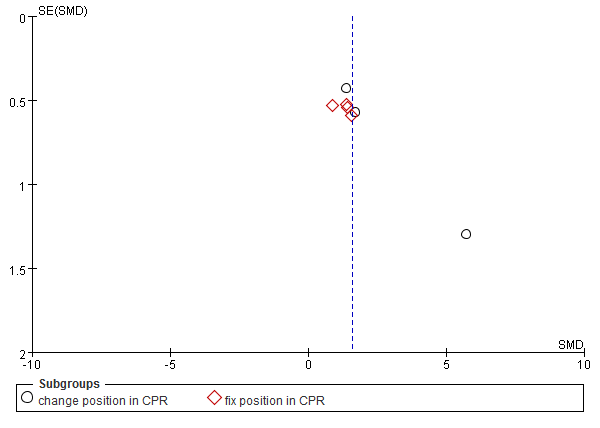

Supplement: Supplementary file 1 — Additional file 1. The funnel plot. [file 13054_2021_3797_MOESM1_ESM.docx]

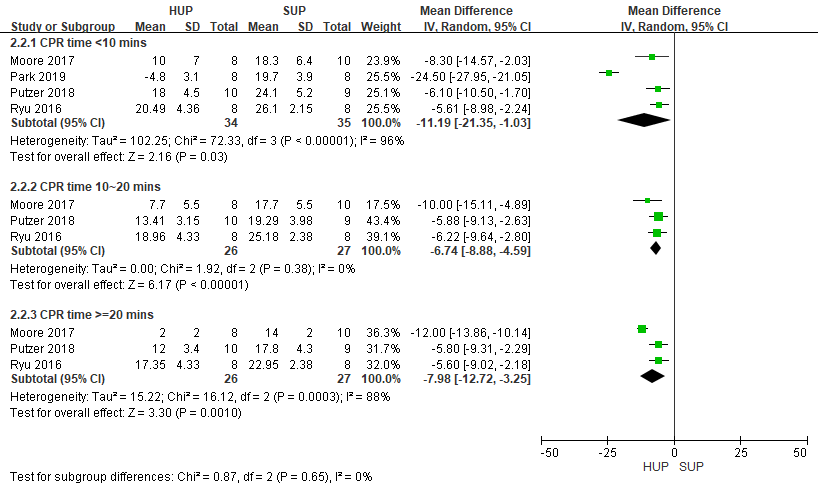
Additional file 2 ICP CPR time

Supplement: Supplementary file 2 — Additional file 2. ICP in different CPR time. [file 13054_2021_3797_MOESM2_ESM.docx]

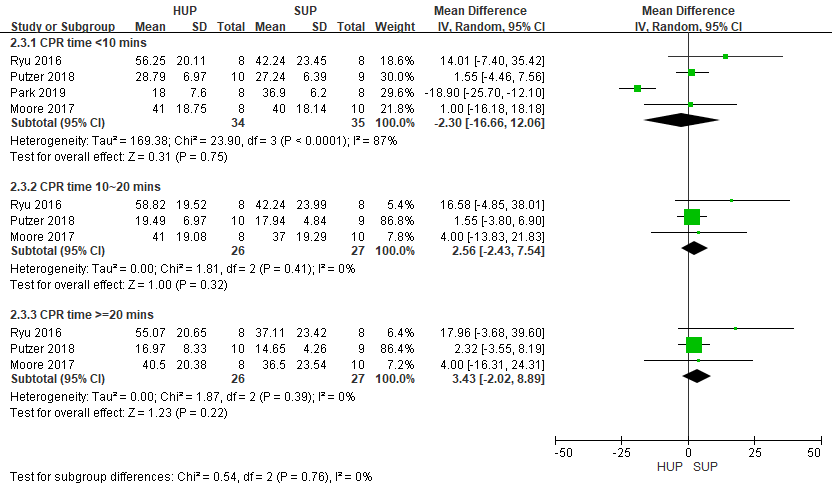
Additional file 3 MAP CPR time

Supplement: Supplementary file 3 — Additional file 3. MAP in different CPR time. [file 13054_2021_3797_MOESM3_ESM.docx]

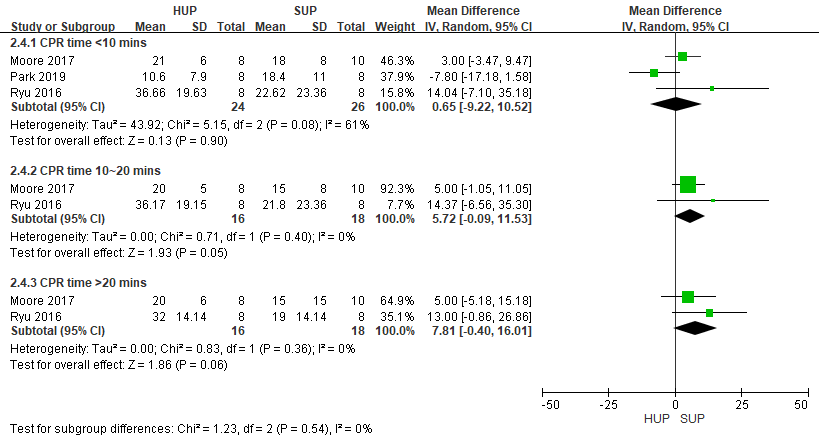
Additional file 4 CoPP CPR time

Supplement: Supplementary file 4 — Additional file 4. CoPP in different CPR time. [file 13054_2021_3797_MOESM4_ESM.docx]
