# Supplementary material for: The effect of the head-up position on cardiopulmonary resuscitation: a systematic review and meta-analysis
Source: Crit Care. 2021 Oct 30;25:376. doi: 10.1186/s13054-021-03797-x (PMC8557496; doi:10.1186/s13054-021-03797-x)
Supplement: Supplementary file 5 — Additional file 5. Automated + ITD versus ACD + ITD CPR. [file 13054_2021_3797_MOESM5_ESM.docx]

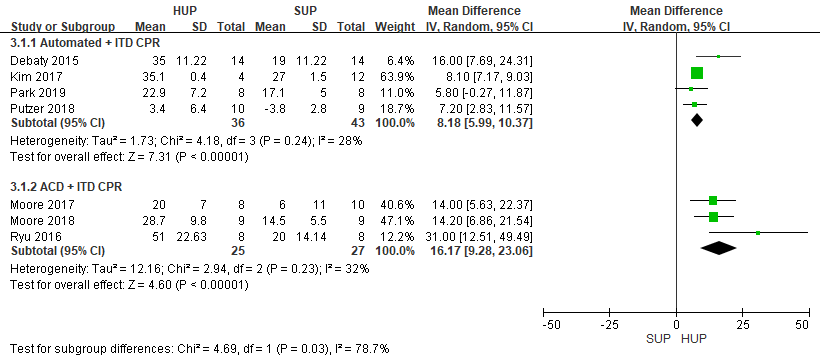


1. CerPP showed higher in ACD + ITD groups


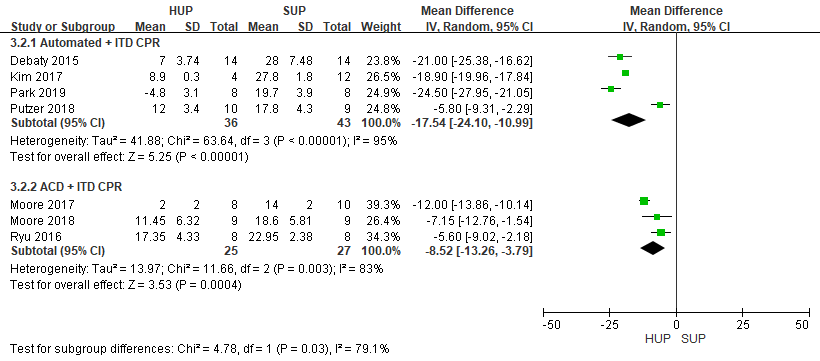


1. ICP was higher in ACD + ITD groups


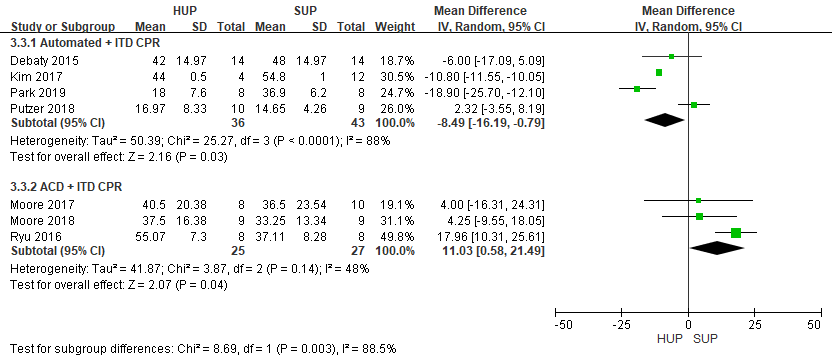


1. MAP increased significantly in ACD + ITD groups


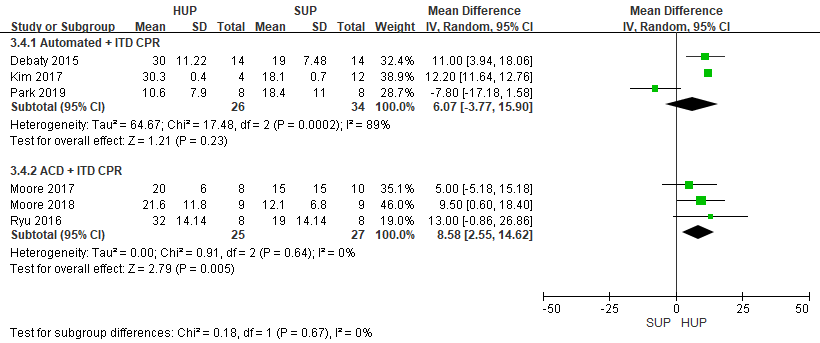


1. CoPP showed similar between two groups
