# Supplementary material for: The effect of the head-up position on cardiopulmonary resuscitation: a systematic review and meta-analysis
Source: Crit Care. 2021 Oct 30;25:376. doi: 10.1186/s13054-021-03797-x (PMC8557496; doi:10.1186/s13054-021-03797-x)
Supplement: Supplementary file 6 — Additional file 6. “Priming” versus “no priming”. [file 13054_2021_3797_MOESM6_ESM.docx]

1.
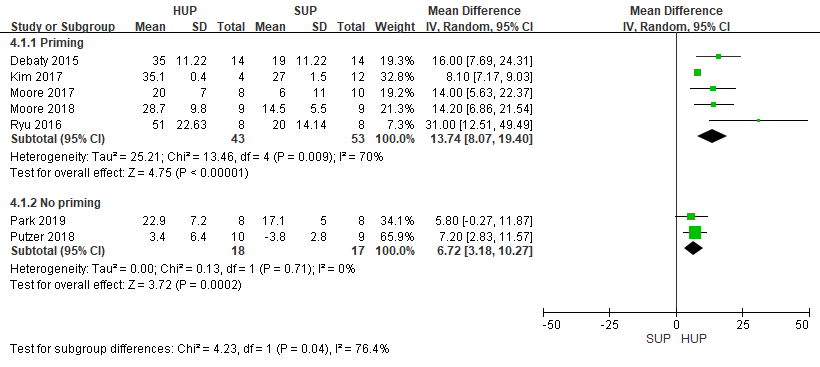
CerPP showed higher in “Priming” group


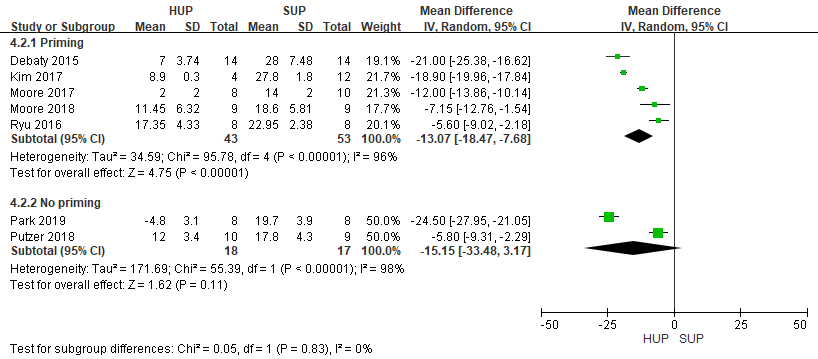


1. ICP was similar in both group


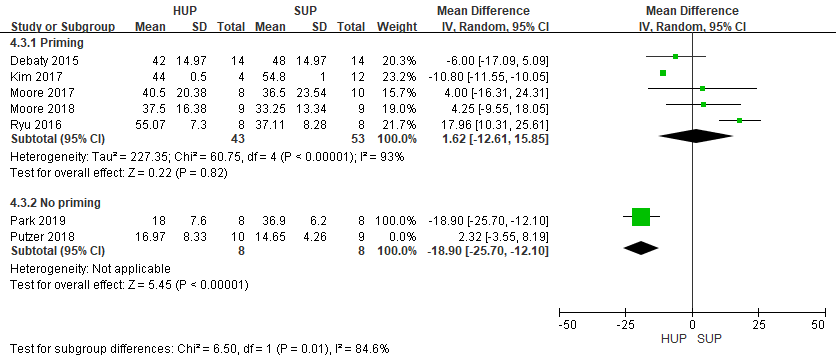


1. MAP was significantly higher in the “priming” group after removing Putzer et al.


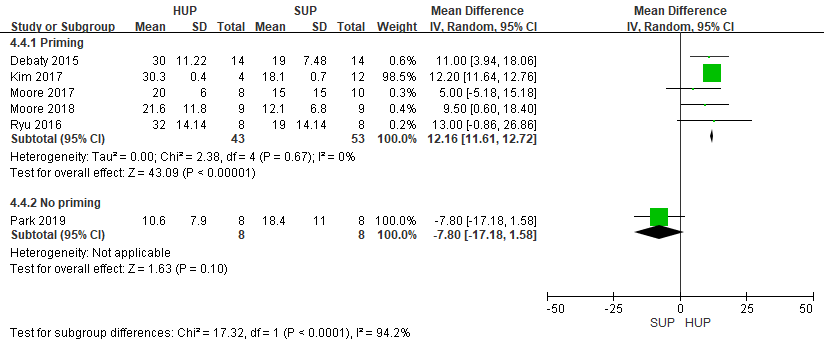


1. CoPP increased significantly in “Priming” group
