# Supplementary material for: The effect of the head-up position on cardiopulmonary resuscitation: a systematic review and meta-analysis
Source: Crit Care. 2021 Oct 30;25:376. doi: 10.1186/s13054-021-03797-x (PMC8557496; doi:10.1186/s13054-021-03797-x)
Supplement: Supplementary file 7 — Additional file 7. “Head/chest up only” position versus “reverse-Trendelenburg” position [file 13054_2021_3797_MOESM7_ESM.docx]

1.
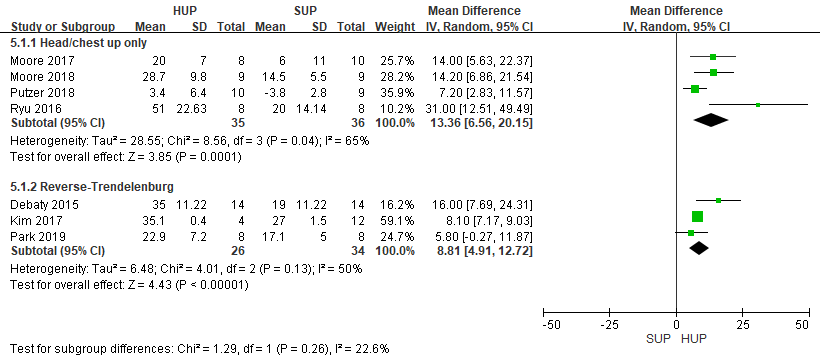
CerPP showed similar in both groups


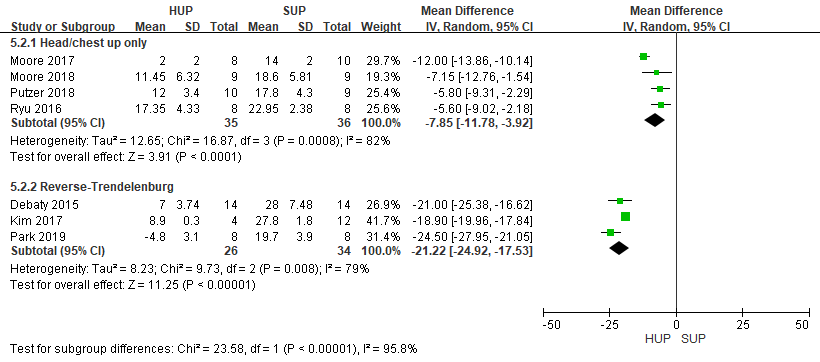


1. ICP was higher in “head/chest up only” position group


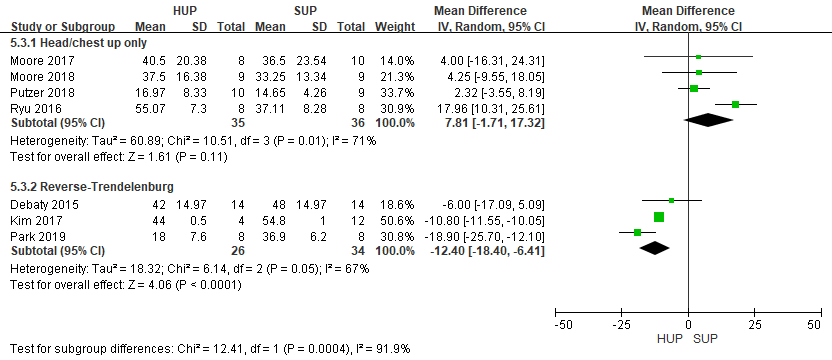


1. MAP showed higher in “head/chest up only” position group


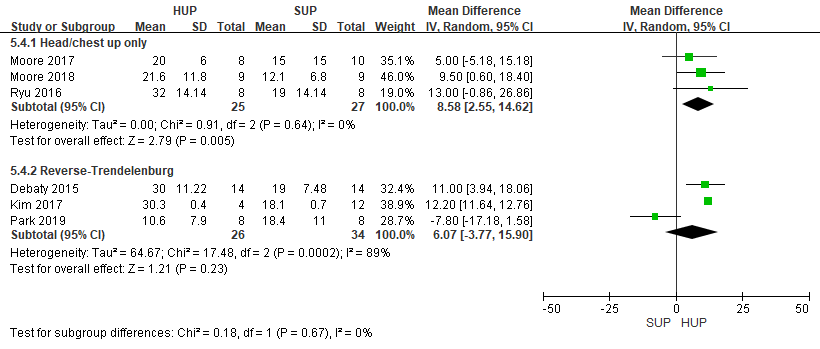


1. CoPP showed similar in both groups
